# Comparison of metastatic castration-resistant prostate cancer in bone with other sites: clinical characteristics, molecular features and immune status

**DOI:** 10.7717/peerj.11133

**Published:** 2021-03-31

**Authors:** Zhengquan Xu, Yanhong Ding, Wei Lu, Ke Zhang, Fei Wang, Guanxiong Ding, Jianqing Wang

**Affiliations:** 1 Department of Orthopedics, The Affiliated Suzhou Hospital of Nanjing Medical University, Suzhou Municipal Hospital, Gusu School, Nanjing Medical University, Nanjing, China; 2 Department of Urology, The Affiliated Suzhou Hospital of Nanjing Medical University, Suzhou Municipal Hospital, Gusu School, Nanjing Medical University, Nanjing, China; 3 Suzhou Vocational Health College, Suzhou, China; 4 Department of Urology, Huashan Hospital, Shanghai, China

**Keywords:** Metastatic castration-resistant prostate cancer (mCRPC), Bone metastasis, RNA sequencing, Bioinformatics analysis

## Abstract

Metastatic castration-resistant prostate cancer (mCRPC) is the lethal stage and the leading cause of death in prostate cancer patients, among which bone metastasis is the most common site. Here in this article, we downloaded the gene expression data and clinical information from online dataset. We found that prostate cancer metastasis in bone is prone to have higher prostate-specific antigen (PSA) and longer time on first-line androgen receptor signaling inhibitors (ARSI). A total of 1,263 differentially expressed genes (DEGs) were identified and results of functional enrichment analysis indicated the enrichment in categories related to cell migration, cancer related pathways and metabolism. We identified the top 20 hub genes from the PPI network and analyzed the clinical characteristics correlated with these hub genes. Finally, we analyzed the immune cell abundance ratio of each sample in different groups. Our results reveal the different clinical characteristics, the immune cell infiltration pattern in different sites of mCRPC, and identify multiple critical related genes and pathways, which provides basis for individualized treatment.

## Introduction

Prostate cancer (PCa) is one of the most common malignant tumors of male genitourinary system, which is the sixth most common cause of cancer-related deaths. It is estimated that there are 914,000 new cases and 255,000 deaths each year (Siegel, Miller & Jemal, 2016; Torre et al., 2016). The onset of prostate cancer not only causes severe health damage to patients, but also brings a serious economic and psychological burden to the family and society. Endocrine therapy-castration treatment (surgery or drugs) or combined androgen blockade is an effective treatment for patients who cannot be cured radically at present. However, drug resistance appears after 12 to 18 months of treatment and develops into castration-resistant prostate cancer (CRPC) or mCRPC with higher severity and mortality (Shen & Abate-Shen, 2010). Therefore, in-depth exploration of the pathogenesis of CRPC and development of new strategies for the prevention and treatment of diseases have become urgent and arduous tasks.

Bone is the most common metastatic site for prostate cancer (Logothetis & Lin, 2005). The metastases are mainly in the pelvis, spine and proximal femur. Orthopedic treatment is often required to deal with the severe pain caused by bone metastases and bone fracture resulted from pathological fractures (Barlev et al., 2010; Shahinian et al., 2005). Even in some patients, bone pain caused by bone metastasis has become the first symptom due to the insidious onset of prostate cancer, which finally can be confirmed by the pathological examination after surgery. In addition to bone, prostate cancer can also metastasize to other organs, like lung, liver, pleura, and adrenals. Different tumor genomic characteristics result in different metastatic sites, while different metastatic sites determine the different tumor microenvironment, especially the local immune status (Binnewies et al., 2018; Choi et al., 2020). All the features above affect the treatment effect and disease prognosis. However, the differences of clinical characteristics, molecular features, and immune status between bone metastatic tumors and other distant metastatic tumors have not been elaborated and studied in depth. Many recent studies have focused on the feature of CRPC especially mCRPC. These researches could help us better understand the mechanisms of this advanced prostate cancer (Abida et al., 2019).

Here in this study, we downloaded the gene expression data and corresponding clinical information of a latest research on mCRPC to compare the clinically specific differences, molecular phenotypes and expression differences, and immune cell infiltration status in prostate lesions at different metastatic sites comprehensively. Our results could help to find out the critical pathways and related genes to provide candidate targets and strategies for individualized treatment.

## Materials and Methods

### RNA-seq data information

RNA-seq expression data with corresponding clinical information of a published dataset (Abida et al., 2019) were download from online dataset cBioPortal for Cancer Genomics website (Websties: http://www.cbioportal.org/index.do) and GitHub website. (https://github.com/cBioPortal/datahub/tree/master/public/prad_su2c_2019) (Cerami et al., 2012).

### Cell lines and cell culture

DU145 and PC-3 cells (ATCC) were maintained in RPMI1640 supplemented with 10% FBS. All cells were added with antibiotic-antifungal solution (100 units / ml penicillin, 0.1 mg / ml streptomycin and 0.25 mg / ml amphotericin B), and under standard cell culture conditions (5% CO2, 95% humidity) Grow at 37 °C as described previously (Wang et al., 2018). All human cell lines have been authenticated using STR profiling within the last three years.

### Real-time RT-PCR

Total RNAs were extracted from cells using TRIzol reagent (Invitrogen). Quantitative real-time PCR was performed using the Bio-Rad CFX96 system, and the relative gene expression was normalized to internal control as gapdh.

### Gene set enrichment analysis (GSEA)

We figured out the biological function gene sets in prostate lesions at different metastatic sites using software GSEA 4.0.3. Gene mRNA expression matrix was imported and enrichment results satisfying P < 0.05 with a false discovery rate (FDR q-val) <0.25 were considered statistically significant.

### Analysis of DEGs

We analyzed the differential expression of RNA-Seq data using EdgeR (an R package) as previously described (McCarthy, Chen & Smyth, 2012; Robinson, McCarthy & Smyth, 2010). Fold change (FC) ≥ 2 or ≤ 0.5; FDR adjusted *P* value <0.05 were the criteria to determine DEGs.

### Functional annotation and pathway enrichment analysis

We divided the DEGs into upregulated and downregulated groups, and did enrichment analysis in Gene Ontology (GO) and Kyoto Encyclopedia of Genes and Genomes (KEGG) to annotate the related genes’ functions. Database for annotation, visualization and integrated discovery (DAVID) website was used to calculate (DAVID version 6.8) (Dennis Jr et al., 2003).

### Integration of protein–protein interaction (PPI) network and module analysis

STRING is an online tool for functional interaction network, which provides a critical assessment and integration of PPI. In this study, we uploaded all the DEGs to the STRING database online for PPI evaluation and integration (Szklarczyk et al., 2015). The cutoff of PPI network was combined score 0.4. Cytoscape was then used to complete module screening by Molecular Complex Detection (MCODE) (Bader & Hogue, 2003).

### Identification of infiltrated immune cells

We identified the infiltrated immune cells using the online analysis tool CIBERSORT, which could estimate the abundance ratio of member cell types in a mixed cell population from gene expression data (Newman et al., 2015). We uploaded the RNA-Seq expression matrix online to obtain an abundance ratio matrix of 22 immune cells. CIBERSORT derives a *p*-value for the deconvolution of each sample. Such *p*-value could provide a measure of confidence in the results, and *p* < 0.05 was considered accurate. Then, only the samples with *P* < 0.05 were selected for the next analysis. Correlation analysis was then performed on the content of 22 immune cells in the selected samples.

### Statistical analysis

The *t*-test was used to compare the data which are normally distributed. One-way ANOVA test with Bonferroni’s multiple comparison tests was used to compare data from multiple groups. Mann–Whitney U test was used to compare the clinical data which are not normally distributed. The Kaplan–Meier method with log-rank test was used for calculating the clinical outcome by Graphpad. A value of P 0.05 was considered statistically significant. All the statistical analyses were conducted with Graphpad and R 3.3.0.

## Results

### Data source and identifying differences in clinical characteristics

To compare the characteristics of mCRPC in bone metastasis with other sites, we downloaded the RNA-seq data of mCRPC tissues and clinical information of the patients. We obtained the clinical information of 423 mCRPC patients in total, of which only 212 had corresponding gene expression data. According to the metastatic site, we divided all the patients into bone metastasis group and other sites group. In the following analyses, we used the clinical information of all 423 patients because of the integrity of the data in clinical aspect. All samples for RNA-seq were from the metastatic lesions as the information indicated. We summarized all the clinical data of the 423 patients in Table 1 and the available clinical data of 212 with RNA-seq data in Table 2.

**Figure 1:**
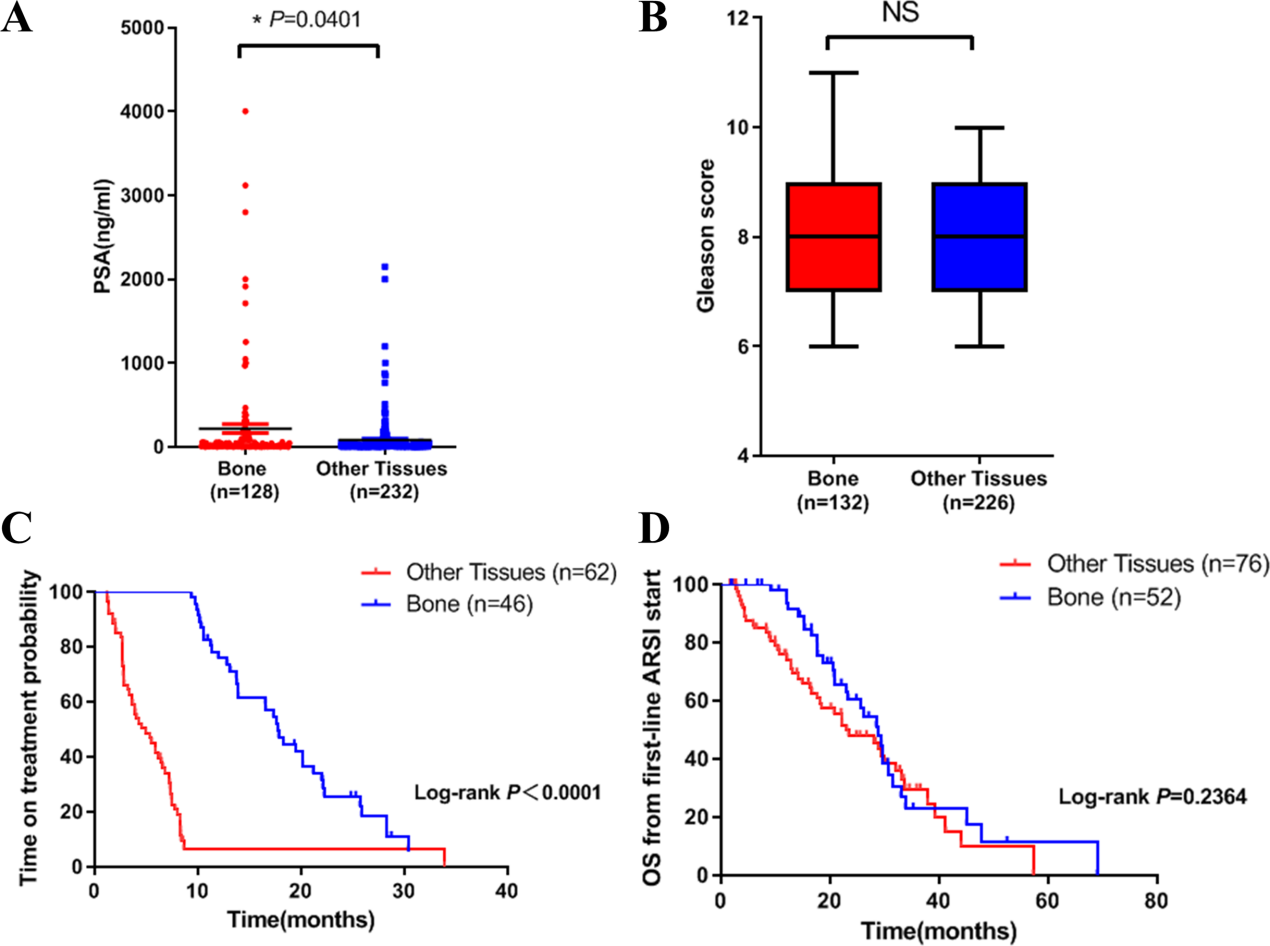
Differences in clinical characteristics between different metastatic sites of prostate cancer. (A) PSA levels in bone metastasis group and other site group. (B) Gleason score in bone metastasis group and other site group. (C) The time on treatment with a first-line ARSI in patients with different metastatic sites. (D) OS time from first-line ARSI in patients with different metastatic sites.

We then compared the clinical characteristics in the two groups. We did the analysis in three aspects: PSA level, Gleason score and clinical outcome. Results showed that PSA level in bone metastasis group was higher (Fig. 1A). There was no difference in Gleason score in both groups (Fig. 1B). In terms of clinical outcome, we analyzed the treatment time and overall survival data after first line ARSI treatment. Results indicated that metastatic site was related to treatment time: patients with bone metastasis had a longer time on treatment with a first-line ARSI (Fig. 1C), but no difference was found in overall survival in both groups (Fig. 1D). Our results indicated that different metastatic sites of mCRPC may have different disease outcome and clinical features.

**Table 1:** Summary of the clinical characteristics of patients with mCRPC.

Characteristics	Case, N (%)
**Age at diagnosis, years**	
≤60	157 (37%)
>60	216 (51%)
Not available	50 (12%)
**Sample Type**	
Adrenal	2 (0.4%)
Bone	155 (36.6%)
Brain	1 (0.2%)
Liver	63 (14.9%)
LN	164 (38.8%)
Lung	7 (1.6%)
Other Soft tissue	29 (6.8%)
Unknown	2 (0.4%)
**Gleason Score**	
≤7	130 (30.7%)
>7	210 (49.6%)
Not available	83 (19.7%)
**PSA at diagnosis**	
<10	135 (31.9%)
10–20	63 (14.9%)
>20	143 (33.8%)
Not available	82 (19.4%)
**Pathology Classification**	
Adenocarcinoma	323 (76.3%)
Small cell	27 (6.4%)
Inadequate for diagnosis or not available	73 (17.2%)
**Abiraterone (ABI) and Enzalutamide (ENZA) Exposure Status**	
Naive	199 (47%)
Exposed	193 (45.6%)
On treatment	19 (4.5%)
Not available	12 (2.9)
**Taxane exposure status**	
Naive	260 (61.5%)
Exposed	142 (33.6%)
Not available	21 (4.9%)

**Table 2:** Summary of the clinical characteristics of patients with mCRPC who had RNA profiles.

Characteristics	Case, N (%)
**Age at diagnosis, years**	
≤60	81 (38.2%)
>60	103 (48.6%)
Not available	28(13.2%)
**Sample type**	
Adrenal	2 (0.95%)
Bone	83 (39.15%)
Liver	27 (12.74%)
Lymph nodes	81 (38.2%)
Other Soft tissue	19 (8.96%)
**Gleason score**	
≤7	64(30.19%)
>7	104 (49.06%)
Not available	44(20.75%)
**PSA at diagnosis**	
<10	49 (23.11%)
10–20	29 (13.68%)
>20	88 (41.51%)
Not available	46(21.7%)
**Abiraterone (ABI) and Enzalutamide (ENZA) exposure status**	
Naive	106 (50%)
Exposed	89 (41.98%)
On treatment	6 (2.83%)
Not available	11(5.19%)
**Taxane exposure status**	
Naive	124 (58.49%)
Exposed	79 (37.26%)
Not available	9(4.25%)

### Differences in molecular aspects

#### GSEA analysis and identification of DEGs

After analyzing the clinical characteristics, we focused on the molecular aspects. After reviewing the clinical data of all patients, we found that some of the patients have ARSI or Taxane exposure. To exclude the influence of treatment, in the parts of molecular and immune cell infiltration analyses below, we only used data of 84 naive mCRPC patients (34 in bone and 50 in other sites, patients without using second-line ARSI (Abiraterone and Enzalutamide) or Taxane) with gene expression data.

**Figure 2:**
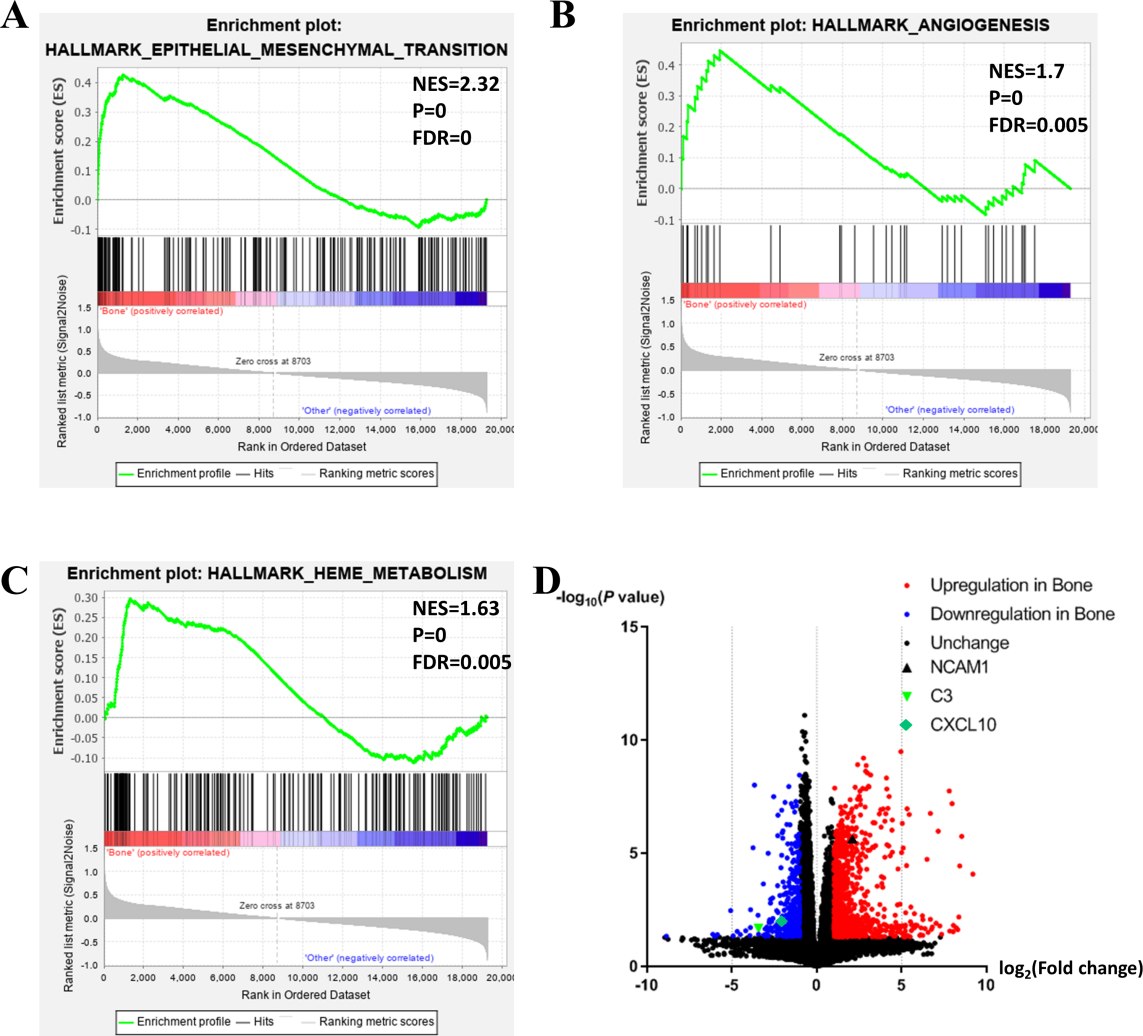
Functional enrichment in different metastatic sites of prostate cancer. (A, B, C) GSEA results of mCRPC bone metastasis, including EMT (A), angiogenesis (B) and heme metabolism (C). (D) Volcano plot for differentially expressed genes. Red and blue symbols classify genes that are up- or down-regulated, respectively. The critical genes NCAM1, C3 and CXCL10 are also indicated.

**Figure 3:**
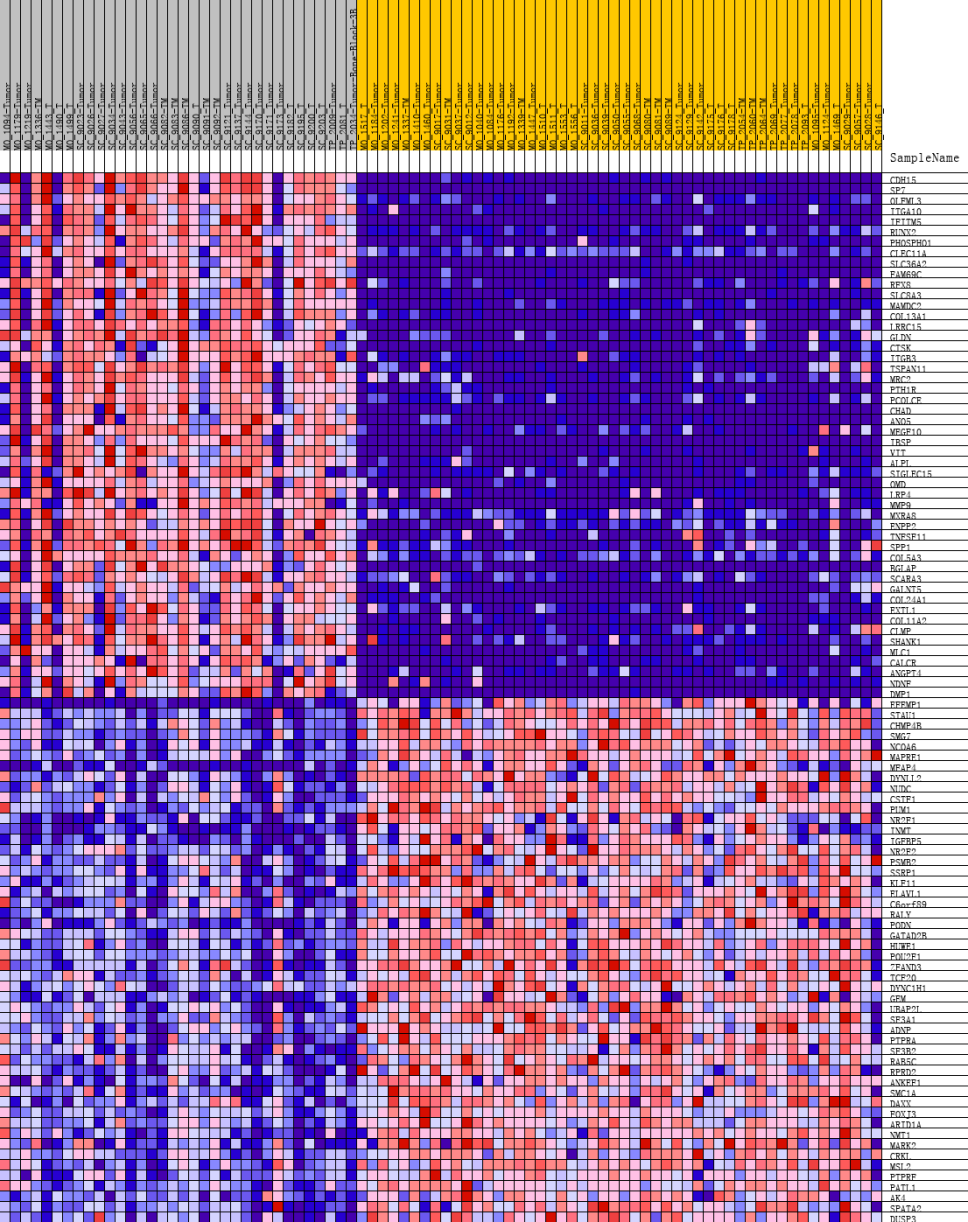
Heat map of the top 100 differentially expressed genes (50 up-regulated genes and 50 down-regulated genes). Red: up-regulation; purple: down-regulation.

To further investigate the molecular difference and find out some evidence, we first analyzed various functional gene sets by the GSEA approach. Results showed that epithelial mesenchymal transition (EMT), heme metabolism, and angiogenesis were significantly enriched in bone metastasis group (Figs. 2A–2C). In further analysis of the molecular difference, we identified the DEGs in the different groups to find out the specific genes upregulated or downregulated in bone metastatic mCRPC samples. Based on the in silico analysis, 1263 genes were identified as DEGs in total. Among all the DEGs, we found 913 upregulated and 350 downregulated in bone metastasis group. The volcano plot of the DEGs is shown in Fig. 2D and the DEGs expression heat map of top 100 genes is shown in Fig. 3.

#### GO and KEGG analyses of DEGs

**Figure 4:**
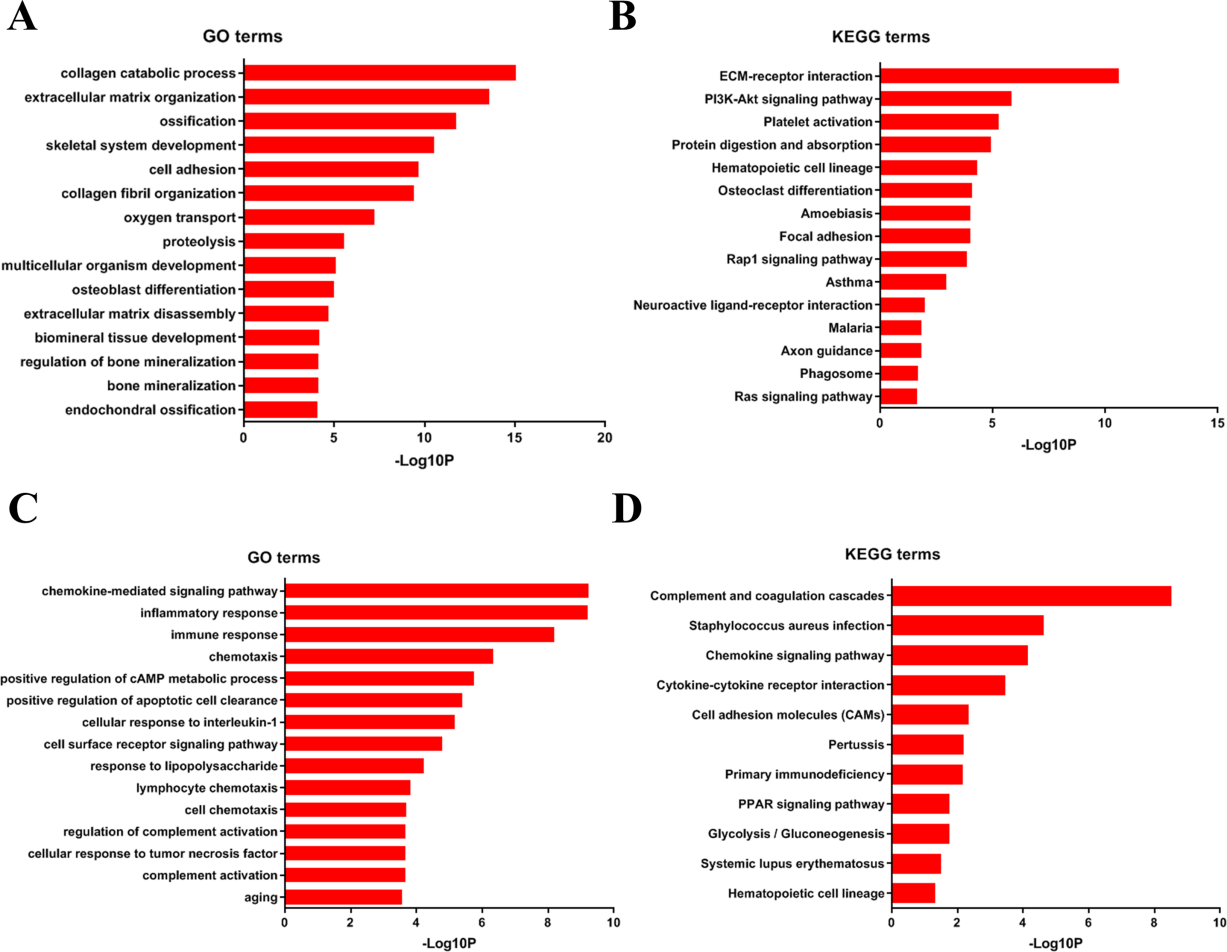
DAVID enrichment results of the differentially expressed genes. (A) The GO enrichment terms of upregulated genes in the bone metastasis group. (B) The KEGG enrichment terms of upregulated genes in the bone metastasis group. (C) The GO enrichment terms of downregulated genes in the bone metastasis group. (D) The KEGG enrichment terms of downregulated genes in the bone metastasis group.

To analyze DEGs at the functional level, we submitted all the upregulated and downregulated DEGs above respectively to DAVID online analysis website. In the upregulated group, the GO analysis of DEGs (Fig. 4A) suggested significant enrichment in collagen catabolic process, extracellular matrix organization, ossification, skeletal system development, cell adhesion, collagen fibril organization, oxygen transport, proteolysis, multicellular organism development, osteoblast differentiation, extracellular matrix disassembly, biomineral tissue development, regulation of bone mineralization, bone mineralization and endochondral ossification. While KEGG pathway analysis showed enrichment mainly in ECM-receptor interaction, PI3K-Akt signaling pathway, platelet activation, protein digestion and absorption, hematopoietic cell lineage, osteoclast differentiation, amoebiasis, focal adhesion, Rap1 signaling pathway, asthma, neuroactive ligand–receptor interaction, malaria, axon guidance, phagosome, Ras signaling pathway (Fig. 4B).

Furthermore, in downregulation group, in GO analysis, DEGs were significantly enriched in chemokine-mediated signaling pathway, inflammatory response, immune response, chemotaxis, positive regulation of cAMP metabolic process, positive regulation of apoptotic cell clearance, cellular response to interleukin-1, cell surface receptor signaling pathway, response to lipopolysaccharide, lymphocyte chemotaxis, cell chemotaxis, regulation of complement activation, cellular response to tumor necrosis factor, complement activation and aging (Fig. 4C). KEGG pathway analysis showed enrichment mainly in complement and coagulation cascades, Staphylococcus aureus infection, chemokine signaling pathway, cytokine-cytokine receptor interaction, cell adhesion molecules (CAMs), Pertussis, Primary immunodeficiency, glycolysis/ gluconeogenesis, PPAR signaling pathway, systemic lupus erythematosus, and hematopoietic cell lineage (Fig. 4D).

### Identification of hub genes and modules from the PPI network

In previous steps, we found that the clinical affairs of bone metastasis were different from tumors in other sites, and then we tried to further explore the interrelationships of DEGs above and to figure out some critical genes. We uploaded all the DEGs online to the STRING database. Ranked by degree, we identified the top 20 genes as hub genes. These genes were ALB, ITGAM, MMP9, COL1A1, C3, IL4, MMP2, COL1A2, SPP1, CCR5, NCAM1, MPO, CCL2, GNG11, CXCL10, SOX9, CCR7, AGT, RUNX2 and TNFSF11. ALB was identified as the top hub gene with the degree of 164 (Table 3).

**Table 3:** Functional roles of the 20 hub genes.

**No.**	**Gene**	**Full name**	**Function**
1	ALB	Serum albumin	Its main function is the regulation of the colloidal osmotic pressure of blood.
2	ITGAM	Integrin alpha-M	Integrin ITGAM/ITGB2 is implicated in various adhesive interactions of monocytes, macrophages and granulocytes as well as in mediating the uptake of complement-coated particles.
3	MMP9	Matrix metalloproteinase-9	May play an essential role in local proteolysis of the extracellular matrix and in leukocyte migration.
4	COL1A1	Collagen alpha-1(I) chain	Type I collagen is a member of group I collagen (fibrillar forming collagen).
5	C3	Complement C3	C3 plays a central role in the activation of the complement system.
6	IL4	Interleukin-4	Participates in at least several B-cell activation processes as well as of other cell types.
7	MMP2	72 kDa type IV collagenase	Ubiquitinous metalloproteinase that is involved in diverse functions such as remodeling of the vasculature, angiogenesis, tissue repair, tumor invasion, inflammation, and atherosclerotic plaque rupture.
8	COL1A2	Collagen alpha-2(I) chain	Type I collagen is a member of group I collagen (fibrillar forming collagen)
9	SPP1	Osteopontin	Binds tightly to hydroxyapatite. Appears to form an integral part of the mineralized matrix.
10	CCR5	C-C chemokine receptor type 5	Receptor for a number of inflammatory CC-chemokines including MIP-1-alpha, MIP-1-beta and RANTES and subsequently transduces a signal by increasing the intracellular calcium ion level.
11	NCAM1	Neural cell adhesion molecule 1	This protein is a cell adhesion molecule involved in neuron-neuron adhesion, neurite fasciculation, outgrowth of neurites, etc.
12	MPO	Myeloperoxidase	Part of the host defense system of polymorphonuclear leukocytes.
13	CCL2	C-C motif chemokine 2	Chemotactic factor that attracts monocytes and basophils but not neutrophils or eosinophils.
14	GNG11	Guanine nucleotide-binding protein G(I)/G(S)/G(O) subunit gamma-11	Guanine nucleotide-binding proteins (G proteins) are involved as a modulator or transducer in various transmembrane signaling systems.
15	CXCL10	C-X-C motif chemokine 10	Chemotactic for monocytes and T-lymphocytes. Binds to CXCR3
16	SOX9	Transcription factor SOX-9	Transcriptional regulator.
17	CCR7	C-C chemokine receptor type 7	Receptor for the MIP-3-beta chemokine.
18	AGT	Angiotensinogen	Essential component of the renin-angiotensin system (RAS), a potent regulator of blood pressure, body fluid and electrolyte homeostasis
19	RUNX2	Runt-related transcription factor 2	Transcription factor involved in osteoblastic differentiation and skeletal morphogenesis.
20	TNFSF11	Tumor necrosis factor ligand superfamily member 11	Cytokine that binds to TNFRSF11B/OPG and to TNFRSF11A/RANK. Osteoclast differentiation and activation factor.

**Figure 5:**
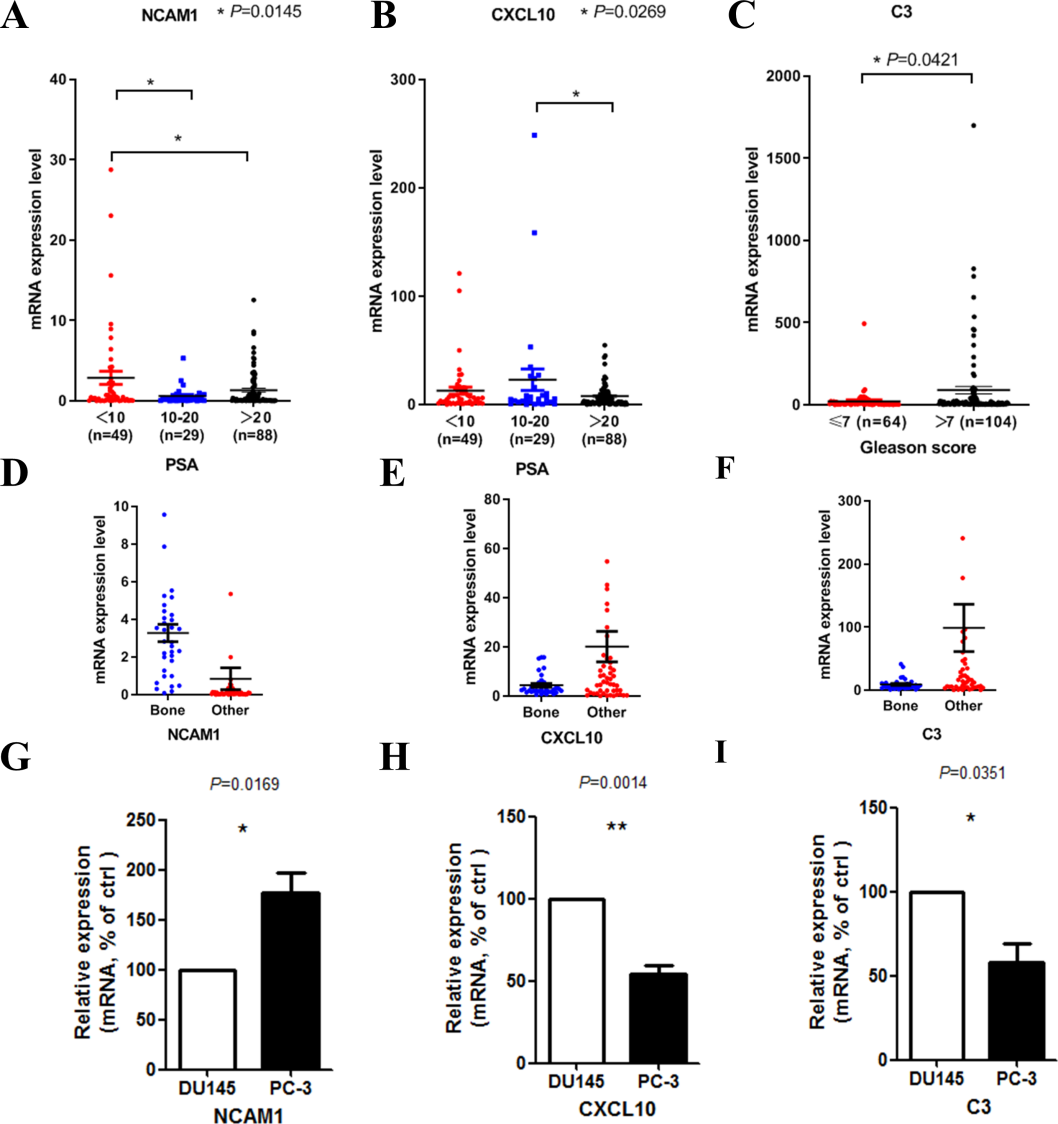
Clinical characteristics analyses of the hub genes. (A) NCAM1 expression level in different PSA level groups (166 samples with both PSA information and gene expression data, one-way ANOVA test with Bonferroni’s multiple comparison test). (B) CXCL10 expression level in different PSA level groups (166 samples with both PSA information and gene expression data, one-way ANOVA test with Bonferroni’s multiple comparison test). (C) C3 expression level in different Gleason score groups (168 samples with both Gleason score information and gene expression data, Mann–Whitney U test). (D–F) The mRNA expression levels of NCAM1 (D), CXCL10 (E) and C3 (F) in the mCRPC dataset. G-I. The mRNA expression levels of NCAM1 (G), CXCL10 (H) and C3 (I) in PC-3 and DU145 cell lines (*t*-test, *n* = 5).

We then tried to find out whether the 20 hub genes above correlated prostate cancer disease progress or outcome. In this part of the research, we hoped to identify some key genes that might play critical roles in disease progression and could be used as potential research targets in the future. In this part, to deal with the analysis of clinical characteristics comprehensively, we used the samples with both clinical information and gene expression data (166 for PSA and 168 for Gleason score). Results indicated that high PSA level correlated with low mRNA expression levels of NCAM1 (Fig. 5A) and CXCL10 (Fig. 5B). Only high expression level of C3 was associated with higher Gleason score (Fig. 5C). We finally identified these genes above, including NCAM1, CXCL10 and C3, as the critical hub genes. Among these genes, NCAM1 expressed higher in bone metastasis group, while CXCL10 and C3 expressed lower in bone metastasis group (Figs. 5D–5F).

To confirm the expression levels of the critical hub genes in bone metastasis group and the other sites group, we used PC-3 and DU145 cell lines to do in vitro experiment. PC-3 is an epithelial cell line from a human prostatic adenocarcinoma metastatic to bone (Kaighn et al., 1979), while DU145 is derived from a central nervous system metastasis (Stone et al., 1978). Our results confirmed that NCAM1 was upregulated in PC-3 cell, while CXCL10 and C3 were downregulated in PC-3 cell (Figs. 5G–5I).

**Figure 6:**
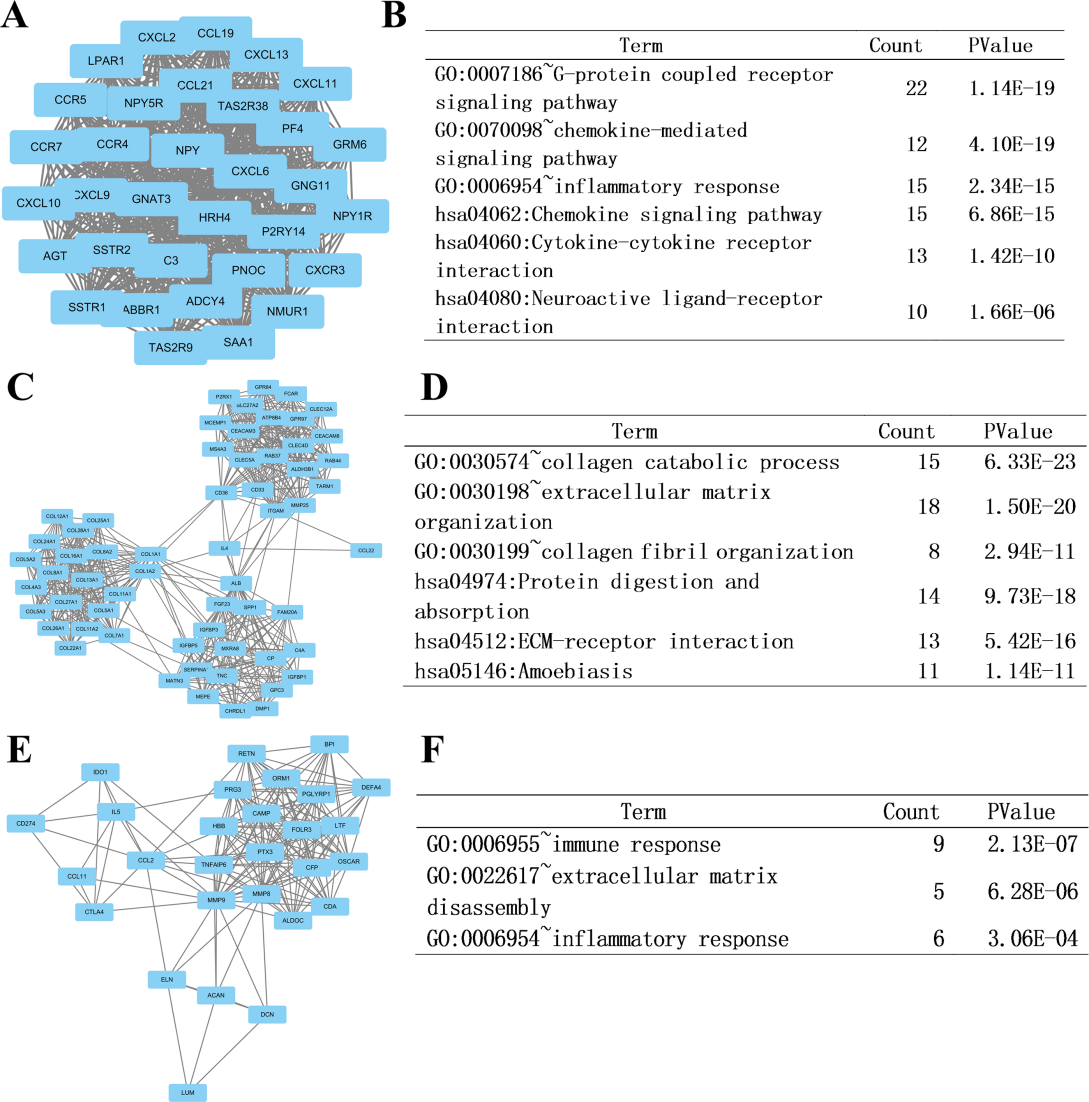
Top three modules from the PPI network and related functional analysis. (A) Results of PPI network of module 1. (B) Results of GO and KEGG analyses of module 1. (C) Results of PPI network of module 2. (D) Results of GO and KEGG analyses of module 2. (E) Results of PPI network of module 3. (F) Results of GO analyses of module 3.

At last, we imported the PPI network above into Cytoscape software to identify modules of genes by MCODE plate, and found out the top 3 modules. To further analyze the modules at the functional level, we did enrichment analysis on the top three significant modules (Fig. 6, respectively). Results showed that functional annotation in module 1 was primarily enriched in G-protein coupled receptor signaling pathway, chemokine-mediated signaling pathway, inflammatory response, chemokine signaling pathway, cytokine-cytokine receptor interaction, and neuroactive ligand–receptor interaction. In Module 2, the analysis showed that the enrichment was mainly related to collagen catabolic process, extracellular matrix organization, collagen fibril organization, protein digestion and absorption, ECM-receptor interaction, and amoebiasis. In module 3, genes were enriched in immune response, extracellular matrix disassembly, and inflammatory response.

### Differences in immune status

#### Identifying immune cell infiltration pattern

**Figure 7:**
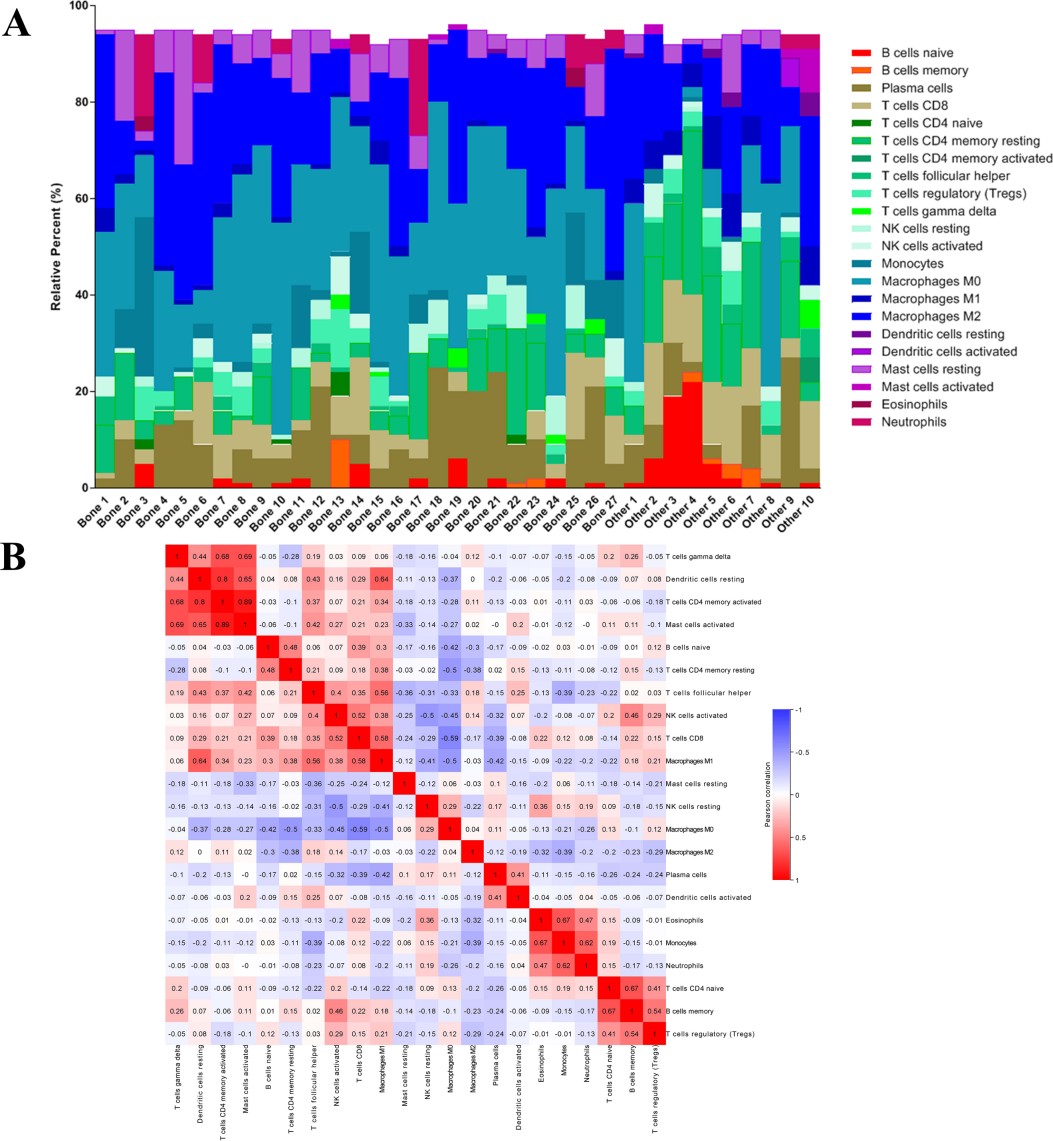
The immune cell infiltration pattern in mCRPC samples. (A) Histogram of the proportion of 22 immune cells in samples P 0.05. (B) The relationship between the abundance ratios of various immune cells. Red represents a positive correlation, and blue represents a negative correlation.

After analyzing the difference in clinial and molecular characteristics, we then analyzed the immune status in the last step. At first, we uploaded the RNA-seq matrix of the 84 patients above online to CIBERSORT website to identify the immune cell infiltration pattern in all these mCRPC tissues. The abundance ratios of 22 immune cells and their correlations were analyzed in the 37 patients with CIBERSORT *p* < 0.05 (Figs. 7A, 7B). T cell CD4 memory activated and mast cells activated, dendritic cells resting were significantly correlated, while macrophage M0 was negatively correlated with T cell CD8. Moreover, we analyzed the OS time from first-line ARSI start and the time on treatment with a first-line ARSI in patients with the abundance ratio of the 22 types of immune cells. Then we divided all the patients into high group and low group according to the abundance ratio of 22 immune cells whose cut-off level was set at the median value, respectively. However, we found no cell type were associated with the clinical outcome significantly (data not shown).

**Figure 8:**
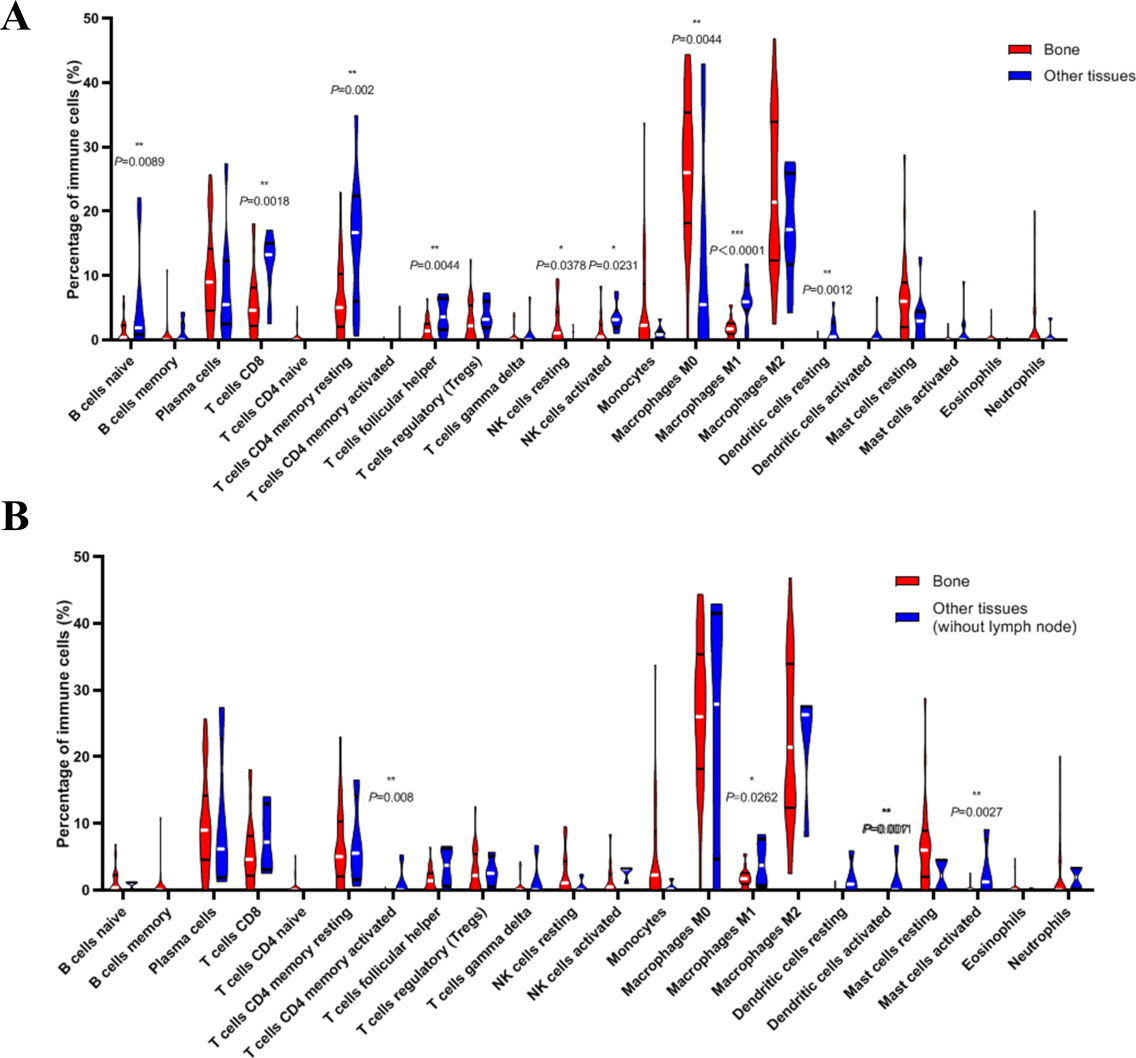
Violin plots of immune cells in bone metastasis tumors and tumors in other locations. (A) Violin plots of immune cells in bone metastasis tumors and tumors in other locations. (B) Violin plots of immune cells in bone metastasis tumors and tumors in other locations without lymph node.

Next, we compared whether the distribution of these 22 immune cells was different in the bone metastasis group and the other sites group. As shown in Fig. 8A, violin plots of immune cells indicated that B cells naïve, T cells CD8, T cells CD4 memory resting, T cells follicular helper, NK cells activated, Macrophages M1, and dendritic cells resting were higher in other sites group, while NK cells resting and Macrophages M0 were distributed higher in bone metastasis group. In further review of the distribution of the metastatic sites, we found some of the ‘other site’ samples are lymph node metastases, which could induce potential bias. To eliminate these potential biases, we also showed the results of immune analysis from bone sites vs other sites (without lymph node) in Fig. 8B. Only T cells CD4 memory activated, Macrophages M1, dendritic cells activated and mast cells activated were distributed higher in other sites group (without lymph node). These results suggest the different immune status in mCRPC bone metastasis group and the other sites group, indicating the potential mechanism underlying the different treatment effect. However, due to the small sample size in the dataset, our results still need further research to confirm in the future.

## Discussion

Metastatic castration-resistant prostate cancer (mCRPC), the fatal stage of prostate cancer, is still the leading cause of cancer related patient death in male (Siegel, Miller & Jemal, 2016). Patients with mCRPC often have lost chance of surgery, and the treatment mostly relies on endocrine therapy and chemotherapy. In recent years, breakthroughs have been made in the research of mCRPC drugs, and a number of second-line ARSI drugs such as abiraterone and enzalutamide have appeared, bringing hope to mCRPC patients. (Scher et al., 2010; Scher et al., 2012). Besides, immunotherapy has become a promising treatment for such advanced malignancies (Inthagard, Edwards & Roseweir, 2019). Because bone metastasis is the major type of prostate cancer metastasis, studying the characteristics of bone metastasis and the differences with other metastatic tumors can help us better understand the pathogenesis of mCRPC and promote individualized treatment strategies.

Here in this study, we comprehensively analyzed the differences between mCRPC in bone and other tissues in clinical, molecular and immune aspects using an RNA-Seq dataset of mCRPC downloaded from GitHub and cBioportal by bioinformatics analysis approaches. In clinical aspect, we found that PSA level in bone metastasis group was higher and that patients with bone metastasis had a longer time on treatment with a first-line ARSI. This indicated that mCRPC in bone metastasis tends to be more malignant but with relatively favorable prognosis, although there was no association with overall survival. Patients with higher PSA level and progression to CRPC may be benefited from early screening for bone metastases, such as radionuclide bone scans. Early detection and treatment of bone metastatic lesions will play a positive role in improving the quality of life of patients and preventing complications such as fractures.

As for molecular aspect, GSEA results showed that EMT, angiogenesis and heme metabolism were enriched in mCRPC bone metastasis. EMT is a crucial step in the progression of prostate cancer, drugs that could reverse or inhibit EMT might be more efficient to the patients with bone metastasis, such as Silibinin (Gazak, Walterova & Kren, 2007; Wu et al., 2010). Accumulated evidence from previous studies has confirmed the importance of angiogenesis in prostate cancer metastasis, especially in bone metastasis, and has validated that inhibition of neovascularization is a promising therapeutic strategy (Lara Jr, Twardowski & Quinn, 2004). Our results add more evidence that angiogenic may be more active in mCRPC bone metastases, indicating that drugs targeted angiogenesis may be more suitable for such patients.

In PPI network analysis, we first analyzed the protein-protein interaction of the DEGs we identified previously. Enrichment analysis results of the top 3 modules revealed that the development of mCRPC bone metastasis was associated with G-protein coupled receptor signaling pathway, chemokine-mediated signaling pathway, inflammatory response, cytokine-cytokine receptor interaction, neuroactive ligand–receptor interaction, collagen catabolic process, extracellular matrix organization, protein digestion and absorption, ECM-receptor interaction, amoebiasis, immune response, and inflammatory response. Some critical pathways and cellular progresses, such as G-protein coupled receptor signaling pathway and collagen metabolism, have been shown to contribute to tumor metastasis or bone metastasis (Costa et al., 2002; Xi et al., 2014). Our results also indicated that other critical pathways and cellular progresses might contribute to the bone metastasis. Drugs or treatment targeted such programs may have specific effects on the inhibition of CRPC bone metastases. Moreover, we also identified 20 hub genes with the highest degree of interaction. ALB (Albumin), which plays a role in regulating plasma osmotic pressure and acts as a carrier protein for a variety of endogenous molecules (including hormones, fatty acids and metabolites) as well as exogenous drugs. Some anti-cancer drugs such as doxorubicin might bind albumin to reach the target organ and play a therapeutic role in prostate cancer (Elsadek et al., 2011). Deep research found that high PSA level correlated with low mRNA expression levels of NCAM1 and CXCL10. In addition, high expression level of C3 correlated with higher Gleason score. These results indicate that the three genes above could be promising candidate oncogenes or tumor suppressors in mCRPC progression in further research.

In immune aspect, we found that the immune cell infiltration pattern in patients with bone metastasis is quite different from those in other locations, which indicated that the different metastatic sites might have different immune cell infiltration pattern. This may result from the different microenvironment, which will affect tumor progression and drug sensitivity in turn. Previous studies have shown that certain inflammatory factors (such as CCL2) can promote prostate tumor growth and bone metastasis by recruiting macrophages (Mizutani et al., 2009). In-depth study of the characteristics of immune cell infiltration in prostate cancer metastases at different locations will help us better understand the local immune characteristics, which also could help us understand its disease progression, and help formulate individualized treatment strategies.

Our study contained some limitations. Our research is a preliminary exploration using RNA-seq data by bioinformatics analysis with some in vitro confirmation. We still need further researches in clinical and molecular biology experiments to confirm our results. The roles of hub genes identified in this study in mCRPC also need to explore. Moreover, sampling adjacent bone healthy tissue will affect the results of RNA-seq, which could be improved by increasing the sample size in the future.

## Conclusions

In conclusion, this study comprehensively compared the clinical features, molecular phenotypes, and immune cell infiltration status of prostate lesions at different metastatic sites. Our results could help to find out the critical pathways and genes related to provide candidate targets and strategies for individualized treatment. However, more clinical and basic researches still are needed in the future.

##  Supplemental Information

10.7717/peerj.11133/supp-1 Supplemental Information 1 Raw RT-PCR data

## Additional Information and Declarations

### Data Availability

The following information was supplied regarding data availability: Raw data are available as a Supplemental File.
